# Sibling method increases risk assessment estimates for type 1 diabetes

**DOI:** 10.1371/journal.pone.0176341

**Published:** 2017-05-16

**Authors:** Hoang V. Lam, Dat T. Nguyen, Cao D. Nguyen

**Affiliations:** 1 Department of Endocrinology, Cho Ray Hospital, Ho Chi Minh City, Vietnam; 2 Department of Science and Technology, Hoa Sen University, Ho Chi Minh City, Vietnam; 3 Department of Business and Information System, Economics University, Ho Chi Minh City, Vietnam; 4 Clinical Analysis and Modelling—Department of Health, Western Australia, Australia; University of Colorado Denver School of Medicine, UNITED STATES

## Abstract

We presented a risk assessment model to distinguish between type 1 diabetes (T1D) affected and unaffected siblings using only three single nucleotide polymorphism (SNP) genotypes. In addition we calculated the heritability from genome-wide identity-by-descent (IBD) sharing between full siblings. We analyzed 1,253 pairs of affected individuals and their unaffected siblings (750 pairs from a discovery set and 503 pairs from a validation set) from the T1D Genetics Consortium (T1DGC), applying a logistic regression to analyze the area under the receiver operator characteristic (ROC) curve (AUC). To calculate the heritability of T1D we used the Haseman-Elston regression analysis of the squared difference between the phenotypes of the pairs of siblings on the estimate of their genome-wide IBD proportion. The model with only 3 SNPs achieving an AUC of 0.75 in both datasets outperformed the model using the presence of the high-risk DR3/4 HLA genotype, namely AUC of 0.60. The heritability on the liability scale of T1D was approximately from 0.53 to 0.92, close to the results obtained from twin studies, ranging from 0.4 to 0.88.

## Introduction

One of the main reasons for disease gene identification is to provide the ability to identify people who are at risk of disease. Thus, a central question for the field is whether validated marker data can be used to discriminate effectively between cases and controls. However, even markers with replicated highly significant odds ratios may be poor classifiers and most variants identified so far confer only small increments in risk and still explain only a small proportion of phenotypic diversity [1]. T1D is a major chronic childhood disease caused by a combination of genetic and environmental influences and genome wide association studies (GWAS) have found over 60 genes to affect the risk of the disease, with the HLA loci having the greatest impact on susceptibility (reviewed in [2, 3]). However, the AUC for risk prediction using multiple identified variants ranges from 0.65 to 0.68 for T1D (see ref. [4] for more details) despite the fact that T1D has a very strong family component with a heritability estimate from 0.4 to 0.8 [5–8].

The association of T1D with alleles at HLA loci, especially the HLA class II genes DR and DQ, is well validated [9]. The highest risk is seen in individuals who are heterozygous HLA-DRB1*03 and HLA-DRB1*04 types. HLA allele typing assists in determining risk for T1D, and in studies to understand the pathogenesis of T1D. It is particularly useful in prevention and intervention trials that test potential preventative treatments in high-risk subjects [10]. However, the high cost of HLA genotyping is a major impost on such large scale programs but is beyond the reach of smaller research groups.

In this study, we presented a cost-effective predictive model that could distinguish T1D status in siblings from multiplex families. Our model can be conducted at birth for early prediction and prevention. Our 3-SNP model can not only prevent mortality, but also decrease morbidity and public health costs.

## Materials and methods

### T1D datasets

We used subjects from the Type 1 Diabetes Genetics Consortium (T1DGC) [11]. A subject was labelled as affected if the subject had documented T1D with onset at 37 years old, had used insulin within 6 months of diagnosis, and had no concomitant disease or disorder associated with diabetes. Most subjects came from families where more than one child was affected, and genotyping and clinical data were also collected for parents and unaffected sibs.

For each family, we randomly selected an affected subject to form a dataset, namely, *probands*. Next, sibs were selected from each family and were paired together with the probands to create 1,253 pairs of *proband-sib*. Then we randomly split the 1,253 pairs into two datasets, namely, a “discovery” dataset of 750 pairs and a “validation” dataset of 503 pairs, subject to the equal proportion of case vs control in each dataset.

### Predictors

We have recently presented a 3-SNP set, namely, *rs2854275*, *rs3104413* and *rs9273363* that could rapidly define the HLA-DR and HLA-DQ types relevant to T1D (see our methods in [12]). We used these SNPs genotyped from the probands as well as theirs sibs to predict the risk of a new sibling at birth to be developed T1D in a multiplex family.

### Risk assessment model

We used a logistic regression model to construct risk prediction models [13]. This method finds the logistic curve that best predicts the risk of disease *P = yes* on the basis of continuous or categorical independents of an observation *G = (g*_*1*_,*…*,*g*_*n*_*)*, formally:

$$Ln\left(\frac{Pr(P=yes|G)}{1\text{-}Pr(P=yes|G}\right)=B_{0}+B_{1}g_{1}+\ldots +B_{n}g_{n}$$ (1)

Logistic regression uses an approach called maximum likelihood estimation to estimate regression coefficients. In this case, the *predictor G* is a 9-dimension vector, including the 3 SNPs genotyped from a proband, the corresponding 3 SNPs genotyped from a sib and 3 binary indicators showing whether or not a genotype from the proband is equal to the corresponding genotype from the sib.

We measured the discriminative accuracy of the predictive models using receiver-operator curve (ROC) analyses [14,15]. The ROC plots the relationship between the true positive rate (TPR or sensitivity) and false positive rate (FPR or 1-specificity) across all possible threshold values that define the disease. The area under the receiver-operator curve (AUC) is the probability that a randomly chosen case will have a higher estimated risk of developing the disease than a randomly chosen control. The AUC ranges from 0.5 to 1, where a higher number implies a better discriminative model between cases and controls. One important feature of the AUC is that it is not dependent on the number of cases or controls tested as described in ref. [16].

### Heritability

Heritability of disease traits is formally defined as the proportion of phenotypic variance in a population attributable to additive genetic factors [17]. Traditionally, heritability is often estimated on the basis of parent-offspring correlations for continuous traits or the ratio of the incidence in first-degree relatives of affected persons to the incidence in first-degree relatives of unaffected persons [5]. In this study, we used the Haseman-Elston regression analysis [18], the simple estimation procedure for *n* sib pairs, of the squared difference between the phenotypes *P*_*i1*_ and *P*_*i2*_ of the *i*^*th*^ pair of siblings on the estimate of their genome-wide IBD proportion $\hat{\pi }$, formally:

$${\left(P_{i1}-P_{i2}\right)}^{2}=\alpha +\beta \hat{\pi }$$ (2)

Because T1D subjects were recruited from different families across worldwide [2,3] the equation (2) is adjusted for stratification using a linear mixed model:

$${\left(P_{i1}-P_{i2}\right)}^{2}=\alpha +\beta \hat{\pi }+u$$ (3)

where *u* is random effect from T1D samples’s regions. The fixed effect coefficient *β* is estimated using the *lme4* package [19] after determining that the genome-wide IBD were distributed normally. We assume that the parents are not inbred so an estimate of the narrow sense heritability is simply:

$${\hat{h}}_{o}^{2}=-\hat{\beta }/(2{\hat{\delta }}_{p}^{2})$$ (4)

where ${\hat{\delta }}_{p}^{2}$ is an estimate of the total phenotypic variance.

To account for ascertainment that generates a much higher proportion of cases in our analyzed samples than in the population, the estimate heritability ${\hat{h}}_{o}^{2}$ on the observed scale case-control study was transformed to that on the liability scale as [17,20]:

$$h_{l}^{2}={\hat{h}}_{o}^{2}\frac{K(1\text{-}K)}{z^{2}}\frac{K(1\text{-}K)}{P(1-P)}$$ (5)

Where K is the population prevalence of T1D disease, *P* is the proportion of case vs. control and $z=e^{-T^{2}/2}/\sqrt{2\pi }$ is the height of the standard normal probability density function at the truncation threshold *T* = *Φ*^*-1*^*(1-K)*.

## Results

### IBD estimate

**Fig 1:**
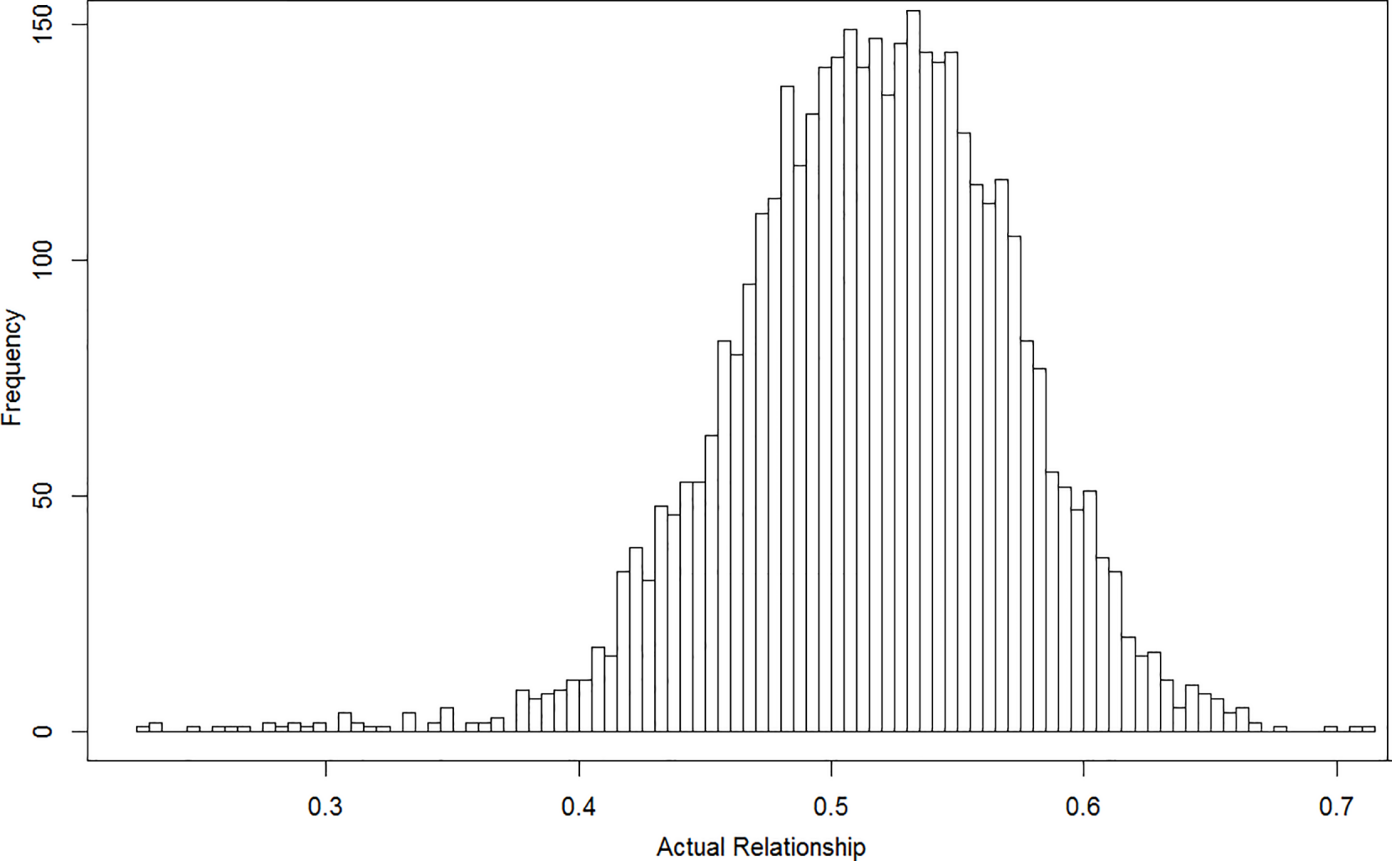
Histogram of the genome-wide additive genetic relationships of full-sib pairs estimated from genetic markers using Plink.

IBD probabilities were calculated using Plink [21]. Fig 1 shows the distribution of the genome-wide additive coefficients. The average proportion of the genome-shared IBD between the sib pairs (the coefficient of additive genetic variance) was 0.516 (standard deviation 0.056), with a range of 0.226–0.715.

### Heritability

We used a non-parametric bootstrap method [22] to calculate the standard error of the heritability. We divided the sib-pair dataset into three subsets, namely affected-affected sib-pairs, affected-unaffected sib-pairs and unaffected-unaffected sib-pairs. From each subset, we randomly selected with replacement 300 sib-pairs to reconstruct a new dataset of 900 samples where the proportion of case vs. control was always fixed at 0.5. We repeated this bootstrap routine 10,000 times to generate 10,000 new different datasets. Table 1 shows the overall *h*^*2*^_*L*_ of T1D ranging from 0.53 to 0.92 depends on different settings of the T1D prevalence *K*. Our heritability estimates using sib-pair methods are closed to the results obtained from twin studies, ranging from 0.4 to 0.88 [5–8]. The R program and IBD data are available in Supporting Information files.

**Table 1:** The heritability on liability scale (*h*^*2*^_*L*_) of T1D estimates using the well-known Haseman-Elston regression analysis.

*T1D prevalence K*	*h*^*2*^_*L*_ *(s*.*e*.*)*
0.003 (general population) 0.005 (general population) 0.01 (siblings of affected probands) 0.03 (siblings of affected probands)	0.53 (0.0017) 0.59 (0.0019) 0.69 (0.0022) 0.92 (0.0029)

### AUC

**Fig 2:**
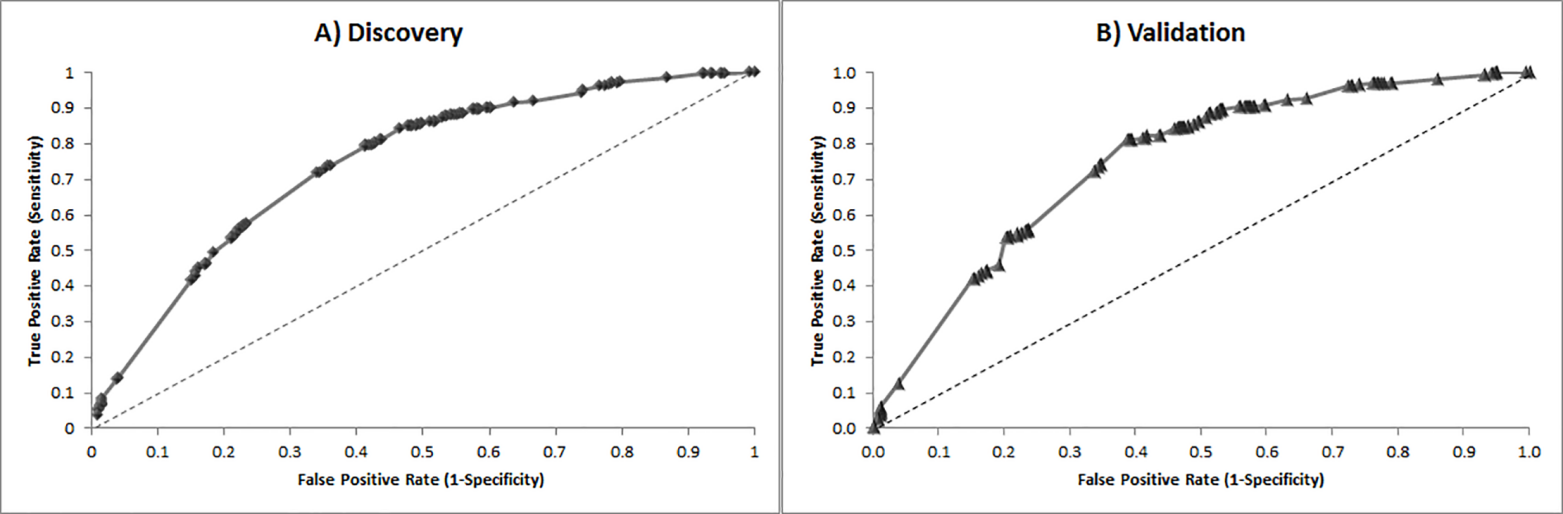
ROC analyses from logistic regression models on *A) T1D’s discovery set n = 750 pairs (1*,*500 samples)* and *B) T1D’s validation dataset n = 503 pairs (1*,*006 samples)*. *Each point represents a test defined by a different logit score*.

The AUC and the corresponding 95% CIs for the sib-pair logistic regression model obtained in the discovery and validation sets are shown in Table 2. The AUC for the model was 0.75 (95% CI, 0.72–0.77) in the discovery set, and when applied to the validation set, the AUC was also 0.75 (95% CI, 0.72–0.78). Thus, the model revealed consistency between the discovery and the validation sets. The overall AUCs in both datasets are far better than those of the model using only the presence / absence of the high risk HLA-DR3/4, namely AUC of 0.61 (s.e. 0.014–0.018). Fig 2 shows the ROC analyses from the sib-pair logistic regression model applied on the two datasets.

**Table 2:** *AUC* on T1D discovery and validation datasets generated from logistic regression models.

Model	Discovery set n = 750 pairs (1,500 samples)	Validation set N = 503 pairs (1,006 samples)
Presence / Absence DR3/4	0.61 (s.e. 0.014) 95% CI (0.59–0.64)	0.61 (s.e. 0.018) 95% CI (0.57–0.64)
3 SNPs *rs2854275*, *rs3104413*, *rs9273363*	0.75 (s.e. 0.013) 95% C.I. (0.72–0.77)	0.75 (s.e. 0.015) 95% C.I. (0.72–0.78)

## Discussion

In the past 15 years, the genetics of common human diseases has been transformed by GWAS. These studies have been a powerful approach to the identification of genes involved in these complex diseases and led to developing predictive genetic tests. The tests using SNPs to predict an individual’s future risk of disease are one of the most appealing early disease prediction methods. Such tests can be conducted at birth and, by use of appropriate prevention strategies, prevent individuals from contracting a disease. These tests have the potential to be the cornerstone of epidemiology and are anticipated to have a large impact on health care (see further reviews in refs [23–25]). It is important to note that ROC curve is a simple and convenient overall measure of diagnostic test accuracy and does not depend on the prevalence of disease in the actual population. However, to measure the performance of a prediction model in clinical settings, the positive predictive value (PPV) and negative predictive value (NPV), which incorporate the disease prevalence in the testing population, are necessary. PPV is the proportion of patients who test positive for the disease who actually have the disease, and the NPV is the proportion of subjects who test negative who are actually free of the disease. Note that, like sensitivity and specificity, the positive and negative predictive values are dependent on the risk score threshold *T* (the logit score in this study). When disease is rare like T1D, the threshold should be selected toward lower left portion of the ROC curve where the sensitivity is small but the specificity is high [13]. For example, when siblings of affected probands are screened, the proposed 3-SNP model achieves the PPVs of 2.7% and 7.8%, and the NPVs of 99.3% and 97.9% for the prevalence of 1% and 3%, respectively. In this case, screening for low prevalence T1D disease is cost effective because the cost of screening is less than the cost of care if the disease is not detected before disease onset. T1D, an autoimmune disease resulting from immune-mediated destruction of the insulin-producing β-islet cells of the pancreas, causes substantial morbidity and mortality and requires life-long insulin treatment. By the time that the disease is detected clinically, the β-cells are almost completely destroyed, and no known treatment can restore them.

Even though preventing or curing type 1 diabetes at risk subjects remain elusive despite effortless and substantial investments in industrialized countries [26,27], diabetes prevention research has been developed rapidly in recent years. In addition to current impressive methods to prevent type 1 diabetes such as metabolic modifications, antigen-specific vaccination, pancreatic transplantation, stimulation of β-cell regeneration, or avoidance of environmental triggers of islet autoimmunity [28], advances in stem cell biology, cell encapsulation methodologies, and immunotherapy will benefit the lives of patients in the end [27,29,30]. Importantly, if early onset diabetes of young children could be identified, screening high risk individuals can stave off or even avoid the short term as well as long term complications of type 1 diabetes. For short term complications, monitoring sugar can prevent new-onset diabetic ketoacidosis, the most severe acute diabetes-related central nervous system complication in young patients [31]. Early monitoring and modification of insulin sensitivity can also hamper diabetic nephropathy, one of the major causes of morbidity and mortality in type 1 diabetes [32,33]. The mortality and morbidity of heart disease are significantly escalated in type 1 diabetes patients compared to the nondiabetic population. An intervention at early stage to achieve glycaemia as close to normal as possible could alleviate and/or delay all of the cardiovascular complications of diabetes in high risk patients [34,35]. Several international projects for diabetes prevention such as DIPP [36], TEDDY [37], TRIGR [38], TrialNet [39] have screened and monitored thousands of newborn infants for HLA-DQB1 allele association with susceptibility to type 1 diabetes. As shown in the results, our proposed method is more accurate and much cheaper than the typical HLA typing.

Genetic factors play a significant role in T1D disease, as indicated by the proportion of explained variance (*h*^*2*^). As the heritability estimates for T1D explain ~90% of the phenotypic variance the GWAS-based predictions can be significantly improved by incorporating many other factors. These include invoking rare variants, structural variants, interaction between genes and environment factors, non-linear interaction between genes and genes, family history conditional on genotype at known loci and signals in non-coding regions [20,23–25].

## Supporting information

S1 File Histogram R. R program for producing histogram of IBD data.
(R) Click here for additional data file.

S2 File Heritability R. R program for calculating t1d h2 using Haseman-Elston regression analysis.
(R) Click here for additional data file.

S3 File Sibling IBD data. Plink IBD probabilities for all sib-pairs studied in this project.
(TXT)

S4 File Sibling IBD data. Plink IBD probabilities for non-affected vs non-affected sib pairs.
(TXT)

S5 File Sibling IBD data. Plink IBD probabilities for non-affected vs affected sib pairs.
(TXT)

S6 File Sibling IBD data. Plink IBD probabilities for affected vs affected sib pairs.
(TXT)
